# Experimental and Metabolic Modeling Evidence for a Folate-Cleaving Side-Activity of Ketopantoate Hydroxymethyltransferase (PanB)

**DOI:** 10.3389/fmicb.2016.00431

**Published:** 2016-03-31

**Authors:** Jennifer J. Thiaville, Océane Frelin, Carolina García-Salinas, Katherine Harrison, Ghulam Hasnain, Nicole A. Horenstein, Rocio I. Díaz de la Garza, Christopher S. Henry, Andrew D. Hanson, Valérie de Crécy-Lagard

**Affiliations:** ^1^Department of Microbiology and Cell Science, University of FloridaGainesville, FL, USA; ^2^Horticultural Sciences Department, University of FloridaGainesville, FL, USA; ^3^Tecnológico de MonterreyCampus Monterrey, Monterrey, Mexico; ^4^Department of Chemistry, University of FloridaGainesville, FL, USA; ^5^Mathematics and Computer Science Division, Argonne National LaboratoryArgonne, IL, USA; ^6^Computation Institute, The University of ChicagoChicago, IL, USA; ^7^Genetics Institute, University of FloridaGainesville, FL, USA

**Keywords:** metabolite repair, side-reaction, *Acinetobacter baylyi*, paralogs, folate biosynthesis

## Abstract

Tetrahydrofolate (THF) and its one-carbon derivatives, collectively termed folates, are essential cofactors, but are inherently unstable. While it is clear that chemical oxidation can cleave folates or damage their pterin precursors, very little is known about enzymatic damage to these molecules or about whether the folate biosynthesis pathway responds adaptively to damage to its end-products. The presence of a duplication of the gene encoding the folate biosynthesis enzyme 6-hydroxymethyl-7,8-dihydropterin pyrophosphokinase (FolK) in many sequenced bacterial genomes combined with a strong chromosomal clustering of the *folK* gene with *panB*, encoding the 5,10-methylene-THF-dependent enzyme ketopantoate hydroxymethyltransferase, led us to infer that PanB has a side activity that cleaves 5,10-methylene-THF, yielding a pterin product that is recycled by FolK. Genetic and metabolic analyses of *Escherichia coli* strains showed that overexpression of PanB leads to accumulation of the likely folate cleavage product 6-hydroxymethylpterin and other pterins in cells and medium, and—unexpectedly—to a 46% increase in total folate content. *In silico* modeling of the folate biosynthesis pathway showed that these observations are consistent with the *in vivo* cleavage of 5,10-methylene-THF by a side-activity of PanB, with FolK-mediated recycling of the pterin cleavage product, and with regulation of folate biosynthesis by folates or their damage products.

## Introduction

**Figure 1:**
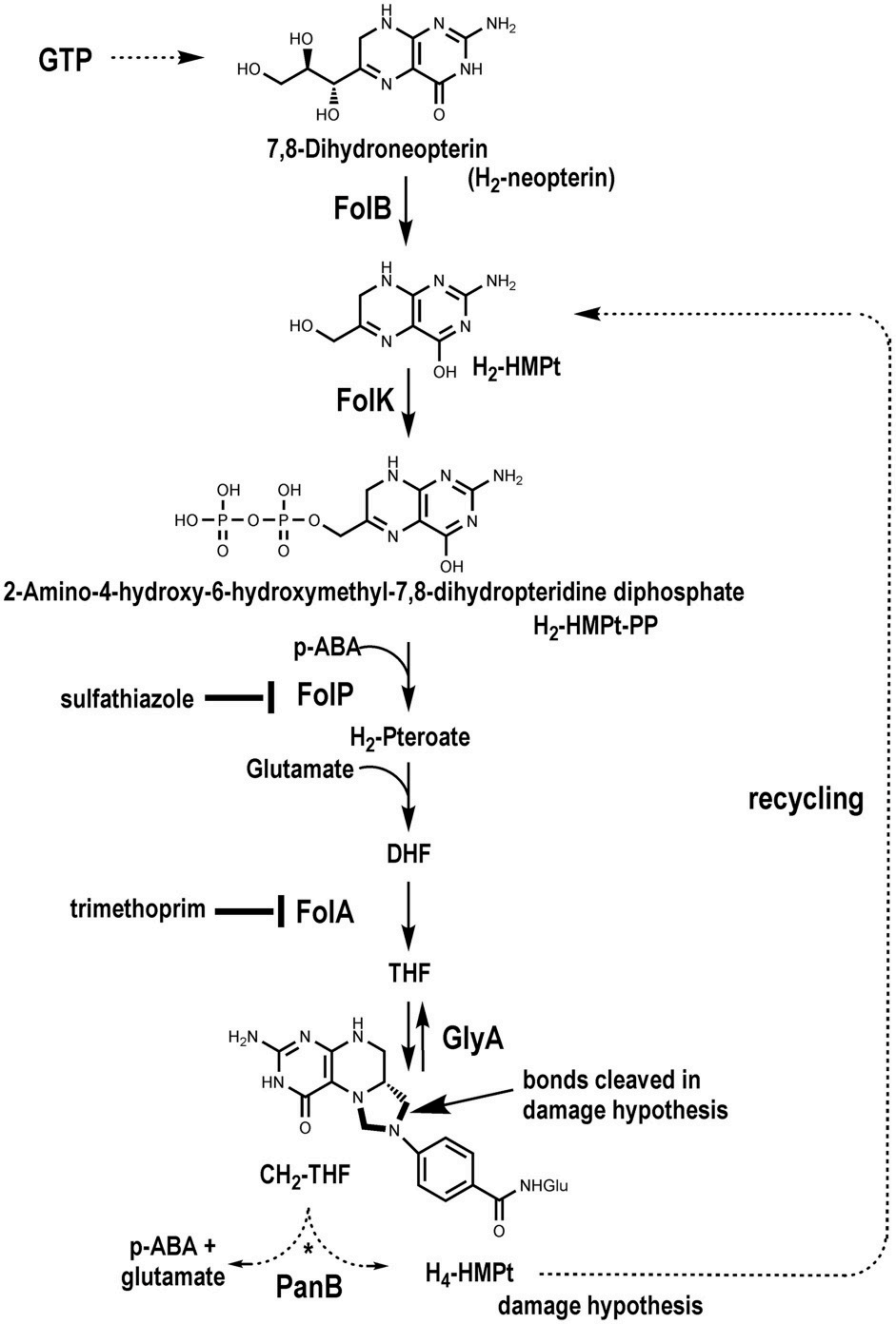
**The classical THF synthesis pathway and a hypothetical folate-cleaving side-reaction mediated by PanB**.

As carriers for one-carbon (C_1_) units in numerous enzymatic reactions, tetrahydrofolate (THF), and its C_1_-substituted derivatives (collectively referred to as folates) are essential in all kingdoms of life. Mammals require a source of THF in the diet but plants and most microbes make THF *de novo*. Although many variations in the THF biosynthesis pathway have recently been discovered (de Crécy-Lagard, 2014), most organisms synthesize THF via the classical pathway summarized in Figure 1 (Cossins and Chen, 1997; Green and Matthews, 2007; Hanson and Gregory, 2011).

The structure of folates, with a reduced pterin moiety linked to *p*-aminobenzoate (pABA) by the C9–N10 bond, makes them inherently unstable. Folates are sensitive at physiological pH to oxidative or photooxidative scission to release the pterin and *p*-aminobenzoylglutamate (pABA-Glu) moieties (Gregory, 1989; Suh et al., 2001; Hanson and Gregory, 2011). The tetrahydropterin ring can also be oxidized to form dihydrofolate (DHF), which can undergo further oxidation to produce scission products. This chemical damage occurs *in vitro* and *in vivo*. Other common sources of damage occur at the level of the pterin precursors of THF, 7,8-dihydroneopterin (H_2_-Neopterin), or 6-hydroxymethyldihydropterin (H_2_-HMPterin), whose side chains are oxidatively labile *in vitro* (Davis et al., 1988; Dántola et al., 2008). This cleavage also occurs *in vivo* as cleavage products such as xanthopterin or 2-amino-4-hydroxypteridine (pterin) have been detected in various bacteria and are sometimes secreted in large amounts (Goto et al., 1965; Forrest and Van Baalen, 1970).

While chemical damage is well-established, the nature and extent of enzymatic damage to THF and other folates are less clear. Bacteria, plants, and mammals have enzymes that can cleave the amide bond in folates, forming glutamate and pteroate moieties, but these hydrolases have wide specificities and evidence that they act on folates *in vivo* is lacking (McCullough et al., 1971; Oe et al., 1983; Bozzo et al., 2008). It is known that mammalian ferritin enhances folate cleavage *in vitro* and *in vivo* (Suh et al., 2000), and an enzyme that cleaves the folate C9–N10 bond has been found in the slime mold *Dictyostelium* (De Wit et al., 1983). Furthermore, the high rates of folate breakdown reported in plants may not be accounted for by chemical instability alone (Orsomando et al., 2006; Hanson and Gregory, 2011). It is conceivable that 6-pyruvoyl-tetrahydropterin synthase-type enzymes, which seem to have wide substrate specificities (Phillips et al., 2012; Seo et al., 2014), can cleave folates. However, another—and unconventional—possibility has not been explored: that enzymes that use folates as C_1_-donors or in other types of chemistry (Waller et al., 2010) mediate folate-cleaving side-reactions.

Although the drain on folate pools from oxidative damage is known to be countered by recycling strategies for the pABA and pterin moieties, these have not been fully elucidated (Quinlivan et al., 2000; Carter et al., 2007; Noiriel et al., 2007a,b; Bozzo et al., 2008; Hanson and Gregory, 2011). Nor is it known how folate biosynthesis responds to folate depletion, although it has been shown that treatment with antifolates causes rapid accumulation of the alarmone ZTP or its precursor ZMP (Bochner and Ames, 1982; Kim et al., 2015).

We present here comparative genomic, genetic, and metabolic modeling evidence that (i) the pantothenate (vitamin B5) biosynthesis enzyme ketopantoate hydroxymethyltransferase (PanB) cleaves 5,10-methylenetetrahydrofolate (CH_2_-THF) as a side-reaction, (ii) that cells repair this damage via the folate biosynthesis enzyme 6-hydroxymethyl-7,8-dihydropterin pyrophosphokinase (FolK), and (iii) that folate biosynthesis is upregulated in response to PanB-inflicted damage.

## Materials and methods

### Bioinformatic analyses

The BLAST tools (Altschul et al., 1997) and resources at NCBI (http://www.ncbi.nlm.nih.gov/) were routinely used. Sequences were aligned using Clustal Omega (Li et al., 2015) or Multalin (Corpet, 1988). Sequence logos were generated using WebLogo3 (http://weblogo.threeplusone.com/) (Crooks et al., 2004) or with the logo comparison tool at http://www.twosamplelogo.org/ (Vacic et al., 2006). Phylogenetic distribution was analyzed in the SEED database (Overbeek et al., 2005). Results are available in the “Folate_biosynthesis” subsystem on the SEED server (http://pubseed.theseed.org/SubsysEditor.cgi?page=ShowSubsystem&subsystem=Folate_Biosynthesis). Physical clustering was analyzed with the SEED subsystem coloring tool or the SeedViewer Compare Regions tool (Overbeek et al., 2014). A subset of FolK sequences from ~1000 representative genomes was extracted from SEED via a perl API query (Disz et al., 2010). An alignment was done using Muscle (http://www.drive5.com/muscle/) (Edgar, 2004) on the command line with default settings. The trees were made with MEGA6 (Tamura et al., 2013) using Maximum Likelihood, Neighbor Joining, and UPGMA methods. The trees were visualized with the ETE2 python package (Huerta-Cepas et al., 2010). The physical clustering information was added to the tree after writing a python code that extracted if *folK* was within 10 coding sequences of the *folB* gene, the *panB* gene or both in a given genome. PDB (www.rcsb.org) (Berman et al., 2000) was used to visualize structures and ligand binding sites.

### Strains and media

Bacteria were grown at 37°C on Luria Bertani (LB) medium (BD Diagnostics Systems) or on M9 minimal medium (Sambrook et al., 1989) supplemented with 0.4% glucose (w/v). Growth media were solidified with 15 g/l agar (BD Diagnostics Systems) for the preparation of plates. Transformations were performed following standard procedures (Sambrook et al., 1989). Chloramphenicol (Cm, 30μg/ml), kanamycin (Kan, 50 μg/ml), ampicillin (Amp, 100 μg/ml), thymidine (dT, 80 μg/ml), and pantothenate (1 μM) were used as appropriate. *E. coli* strains used in this study included MG1655 (*Coli* Genetic Stock Center), BW25113 (Baba et al., 2006), and C600Δ*folK*::tet (Jonsson and Swedberg, 2005). The BW25113 Δ*panB*::kan mutant was obtained from the Keio collection (Baba et al., 2006) and confirmed to be auxotrophic for pantothenate. *E. coli* GC10 (GeneChoice) was routinely used for cloning.

### Antifolate sensitivity

*E. coli* strains MG1655 and BW25113 were each transformed with pCA24N::*panB* and pBAD33. The *panB* overexpression plasmid (pCA24N::*panB*) was obtained from the ASKA collection of *E. coli* clones (Kitagawa et al., 2005) and was confirmed to complement the pantothenate auxotrophy of the BW25113 Δ*panB*::kan strain. pBAD33 encoding chloramphenicol resistance was used as an empty vector control (Guzman et al., 1995). Transformants were selected on LB with Cm. Three colony-purified isolates from each transformation were selected for further analysis. The isolates were tested for sensitivity to folate inhibitors by the Kirby-Bauer Method (Bauer et al., 1966). Cultures were grown in 5 ml M9-glucose medium with chloramphenicol overnight at 37°C with shaking. The following day, the strains were diluted 1:500 in fresh M9-glucose medium and 5 ml of each culture was poured onto M9-glucose agar with added chloramphenicol and IPTG (0.1 mM). The liquid was evenly distributed across the plate and the unabsorbed liquid was removed by pipette. Filter disks were placed on top of the agar, and 20 μl of either 50 or 100 μg/ml of trimethoprim, or 50 or 100 μg/ml of sulfathiazole antibiotics were spotted onto each disk. The plates were incubated for 18 h at 37°C and the diameters of the resulting zones of inhibition were measured.

Sensitivity to antifolates was also measured by dilution drops onto various concentrations of antibiotic. Cultures were grown in 5 ml M9-glucose medium with chloramphenicol overnight at 37°C with shaking. The following day, the strains were diluted 1:20 in fresh M9-glucose medium and grown at 37°C with shaking until the cultures reached approximately A_600_1.0. The cultures were normalized to an A_600_1.0 and serially diluted in 10-fold increments. Ten microliter of each dilution was spotted onto M9-glucose agar plates with added chloramphenicol and IPTG (0.1 mM) with sulfathiazole (0–1 μg/ml) or trimethoprim (0–1.5 μg/ml). Plates were incubated at 37°C overnight. To test the effect of Δ*panB* on sensitivity to folate inhibitors, the same assay was performed with wild-type BW25113 and BW25113 Δ*panB*::kan with the exception that chloramphenicol was not added to the media and all minimal media contained 1 μM pantothenate.

### Folate gene expression analysis

Expression levels of *folB, folE, folK*, and *panB* were analyzed in MG1655 harboring either empty vector pBAD33 or the *panB* overexpression plasmid pCA24N::*panB*. Three biological replicates of each were grown overnight in M9-glucose medium with chloramphenicol at 37°C with shaking. The following day the cultures were diluted 1:100 in fresh M9-glucose medium with chloramphenicol and grown at 37°C with shaking. IPTG was added to 0.2 mM when the culture was at A_600_ 0.2 and the induced cultures were harvested at A_600_1.0. RNA was extracted from each culture with the QIAGEN RNeasy Protect Bacteria Mini Kit, the concentration was determined by NanoDrop spectrophotometer, and RNA was stored at −80°C. cDNA was synthesized from 300 ng RNA using the iScript Select cDNA synthesis kit (Bio-Rad) using the provided random primer mix. Quantitative PCR was performed using the MyiQ2 Real Time PCR Detection System (Bio-Rad) using iTaq Universal SYBR Green Supermix (Bio-Rad). Amplification parameters were 95°C for 3 min followed by 40 cycles of 95°C for 10 s, 59°C for 30 s with fluorescence measurement, followed by the establishment of a melting curve. Data were analyzed with the IQ5 software (Bio-Rad) and the Bio-Rad Expression Macro. Expression was normalized against two reference genes: *rssA* (b1234) and *rpoA* (b3295). All primers used for the RT-PCR are listed in Table S1.

### Cloning of *folK*1 and *folK*2 and complementation of Δ*folK*

ACIAD3062 (*folK*1) and ACIAD2407 (*folK*2) were PCR-amplified with primers ACIAD3062-xbaI-5 (5′-CTA CTCTAGAATGAGCATAACCACCTATAT CG-3′) and ACIAD3062-SalI-3 (5′-CATTGTCGACCGCGT TATTTTTCAACCCAG-3′) and ACIAD2407-xbaI-5 (5′-CAT TTCTAGATTGAACGCCAACGCCACAATTTT TG-3′) and ACIAD2407-SalI-3 (5′-CAATGTCGACGAATT ATGATGAAGAAATCAC-3′), respectively. *Acinetobacter baylyi* ADP1 genomic DNA was used as the template for PCR. PCR products were digested with *Xba*I and *Sal*I, purified with the Zymo Clean and Concentrator-5 kit, and ligated into *Xba*I/*Sal*I-digested pBAD24. The ligation mixtures were used to transform *E. coli* GC10 and transformants were selected on LB with ampicillin. pBAD24::*folK*1 and pBAD24::*folK*2 were confirmed by sequencing and the plasmids were renamed pJJT115 and pJJT107, respectively.

### Complementation of *E. coli* Δ*folK*

pJJT115, pJJT107, or pBAD24 were transformed via electroporation into *E. coli* C600 Δ*folK*::tet. The cells were recovered in 1 ml of LB plus dT for 1 h at 37°C. After recovery, the cells were washed once with 1 ml LB without dT to remove dT, and suspended in 1 ml LB. One hundred microliter of the transformation reaction was spread onto LB with ampicillin containing either 0.2% arabinose (w/v) or dT. If the *folK* gene on the plasmid complements the Δ*folK*, the colonies appear after overnight growth at 37°C on LB with ampicillin and arabinose without dT.

### Pterin and folate analyses

For pterin and folate analysis experiments, a *panB* expression plasmid was constructed as follows. Th*e panB* gene was amplified from genomic DNA of strain W3110 by PCR using primers EcPanBp19Fwd (5′-CTA GAAGCTTATGACAGGAAACAGCTATGAAACCGACCACCATCT C-3′) and EcPanBp19Rev (5′-GATCGAATTCTTAATGGA AAC-TGTGTTCTTCG-3′), and cloned into the *Hind*III and *Eco*RI sites of pUC19 (Yanisch-Perron et al., 1985) to give pUC19::*panB*, which was verified by sequencing. To validate the functionality of the expressed gene of the pUC19::*panB* plasmid, the BW25113 Δ*panB*::kan mutant, which is auxotrophic for pantothenate, was transformed with pUC19::*panB* plasmid. Transformants were able to grow on minimal medium in absence of pantothenate, restoring the growth of the mutant. For pterin analysis of the Δ*panB*::kan mutant, three independent colonies of each strain (BW25113 wild type and Δ*panB*::kan mutant) were grown on M9 glucose (0.4%) and 10 μM pantothenate. Cells were harvested when A_600_ reached 1.0. For *panB* overexpression analyses, the strains (wild type MG1655 harboring the vector pUC19 or containing *panB*) were grown on M9 glucose (0.4%) with added Amp. IPTG (0.5 mM) was added at A_600_ of 0.3. Three independent colonies for each were cultivated. Only the cultures overexpressing *panB* had filaments (cell lysis) after adding IPTG (repeated twice independently). Cells were harvested when A_600_ reached 1.0. Protein contents of harvested bacterial cells were determined using the Pierce bicinchoninic acid (BCA) assay kit. Pterins were extracted, converted to their oxidized forms, and analyzed by HPLC with fluorometric detection as described (Pribat et al., 2010). Folates were extracted and analyzed by HPLC with electrochemical detection as described (Pribat et al., 2010; Srivastava et al., 2011).

### Modeling

A kinetic model of the THF biosynthesis pathway was constructed manually in the Copasi Biochemical System Simulator software (Hoops et al., 2006). Reaction stoichiometry was obtained from KEGG (Kanehisa and Goto, 2000) and kinetic parameters were obtained from BRENDA (Chang et al., 2015). Thermodynamic parameters were computed for all reactions using the group contribution method (Jankowski et al., 2008). Kinetic and thermodynamic parameters were used to evaluate whether each model reaction was reversible or irreversible, and saturated or unsaturated; they were not used directly as they did not replicate the experimentally observed metabolite concentrations and fluxes. The actual kinetic parameters in the model were selected to fit experimentally measured fluxes and metabolite concentrations. In addition to the last four steps of THF biosynthesis, the main CH_2_-THF synthesizing reaction (GlyA), and the proposed PanB side reaction, the model included exchange reactions that simulate the production of H_2_-HMPt and pABA by reactions outside the selected scope of the model. Similarly, exchange reactions were added to simulate the continuous dilution of THF and CH_2_-THF that occurs during cell growth (see Figure S4). The H_2_-HMPt and pABA exchange reactions produce these compounds at a fixed rate of 1.66e-7 mmol ml^−1^ cytoplasm s^−1^, which is equal to the total THF synthesis rate required to counter growth-associated dilution computed in wild-type cells based on measured growth rates. The exchange reactions for consumption of THF and CH_2_-THF were modeled as saturating irreversible enzymatic reactions, with *V*_max_ and *K*_m_ values selected to ensure that steady state concentrations for THF and CH_2_-THF match experimentally measured values. THF consumption fluxes cannot be fixed like the H_2_-HMPt and pABA production fluxes, as this results in an overly constrained model. The model did not include folate polyglutamylation because this is peripheral to the chemistry of the reactions of interest. Nor did it explicitly include hydrolysis of pABA-Glu to pABA and glutamate (Carter et al., 2007), which is also peripheral. Additionally, the reactions included in our model involved seven metabolites that are prevalent in numerous metabolic pathways: ATP, AMP, ADP, phosphate, serine, glycine, and pyrophosphate. We held the concentrations of these metabolites in our model at fixed physiological levels taken from the literature (Kukko and Heinonen, 1982; Kukko-Kalske et al., 1989; Rao et al., 1993; Amin and Peterkofsky, 1995; Bennett et al., 2009), as we expect other pathways that fall outside the scope of our model to be responsible for maintaining these metabolites at their physiological concentrations. All data and parameters relating to model compounds and reactions are available in Tables S2 and S3, respectively. We also provide the model data in Copasi format for the three variations of the model that were applied in this work: (i) wild-type (Additional File 1); (ii) PanB overexpression (Additional File 2); and (iii) PanB overexpression and THF regulation (Additional File 3). Finally, all three models were deposited in BioModels (Chelliah et al., 2015) and assigned the identifiers MODEL1602280001, MODEL1602280002, and MODEL1602280003, respectively.

### Model parameter sensitivity analysis

A sensitivity analysis was performed on the kinetic model to identify which parameters were most significant in governing model behavior. We altered model parameters around their currently defined values, rerunning the model after each alteration, and identifying the changes in predicted steady-state fluxes and concentrations. Many parameters in the model had little impact on overall model behavior. These included the kinetic constants for the FolK, FolP, FolC, and FolA reactions, and nearly all of the fixed metabolite concentrations (see previous section). Increasing the kinetic constant in the mass-action kinetic equation will increase the instantaneous flux of the associated reaction, but then the substrates of the reaction are consumed faster than they are produced, causing a decline in substrate concentration, and restoring flux to its original steady-state value. In this case, the steady-state flux through the Fol[KPCA] reactions will be the flux entering the pathway via the H_2_-HMPt production reaction, which we call *v*_*net*_, plus the flux recycled by the proposed PanB reaction, which we call *v*_*PanB*_. Similarly, increasing an intermediate concentration will temporarily boost the flux of the reaction consuming the metabolite, but then flux will relax to the same steady state value as the metabolite is consumed faster than it is produced.

The model was also insensitive to the saturation state of the FolK, FolP, FolC, FolA, and PanB reactions. All of these reactions were modeled as completely unsaturated reactions, essentially following mass action kinetics. We chose mass action kinetics for these reactions, as opposed to unsaturated Monod kinetics, because this reduces the number of parameters, simplifies the model, and produces equivalent behavior when the enzyme is in a completely unsaturated state. However, we did determine that modeling these reactions as saturated enzymatic reactions results in similar behavior and steady states so long as the *v*_*max*_ values exceed the net steady-state flux that these reactions must achieve (*v*_*net*_ + *v*_*PanB*_).

In contrast, the kinetic parameters on the GlyA reaction exercised a large degree of control over the ratio of steady-state concentrations for THF and CH_2_-THF. The ratio and magnitude of these rate constants dictated how close this reaction was to equilibrium, as well as what the equilibrium ratio of THF and CH_2_-THF would be. The total steady-state concentration of THF and CH_2_-THF was controlled by the value of *v*_*net*_ and the kinetic parameters on the drain reactions for THF and CH_2_-THF. The THF and CH_2_-THF drain reactions represent the consumption of these compounds during cell growth and dilution. Because the value of *v*_*net*_ was fixed, the only adjustable parameters governing the total THF and CH_2_-THF concentration were the kinetic parameters on the drain reactions. These parameters were adjusted to ensure that the total concentration of THF and CH_2_-THF predicted by the model matched experimental data under all conditions studied. Given these constraints, these kinetic parameters were not flexible. Rather, only a single set of values could be found to produce the optimal fit to the experimental data.

The other key parameters in the model are the kinetic constants on the PanB reaction, which control the steady state concentration of H_2_-HMPt, as well as the magnitude of the recycle flux back to H_2_-HMPt. This flux controls the dynamic response of the folate pathway intermediates, including the initial dip that occurs in the H_2_-HMPt concentration immediately following the induction of the PanB enzyme, which we have proposed as the cause of the overall induction of the folate pathway. We tuned the kinetic parameters on the PanB reaction in our model to fit the experimentally measured concentrations for H_2_-HMPt. We currently model this reaction as unsaturated following irreversible mass action kinetics, but behavior would be similar if the reaction followed saturated Monod kinetics.

## Results

### Comparative genomics predicts a functional association between *panB* and *folK* genes

Inspection of the current “Folate biosynthesis” subsystem in the SEED database (Overbeek et al., 2005) revealed that 14% of the ~11,400 genomes analyzed contain two copies of the gene encoding 6-hydroxymethyl-7,8-dihydropterin pyrophosphokinase (*folK*). This observation had already been made when the subsystem was first constructed with far fewer genomes (de Crécy-Lagard et al., 2007), but no explanation for the role of the second copy of the *folK* gene was proposed at that time.

**Figure 2:**
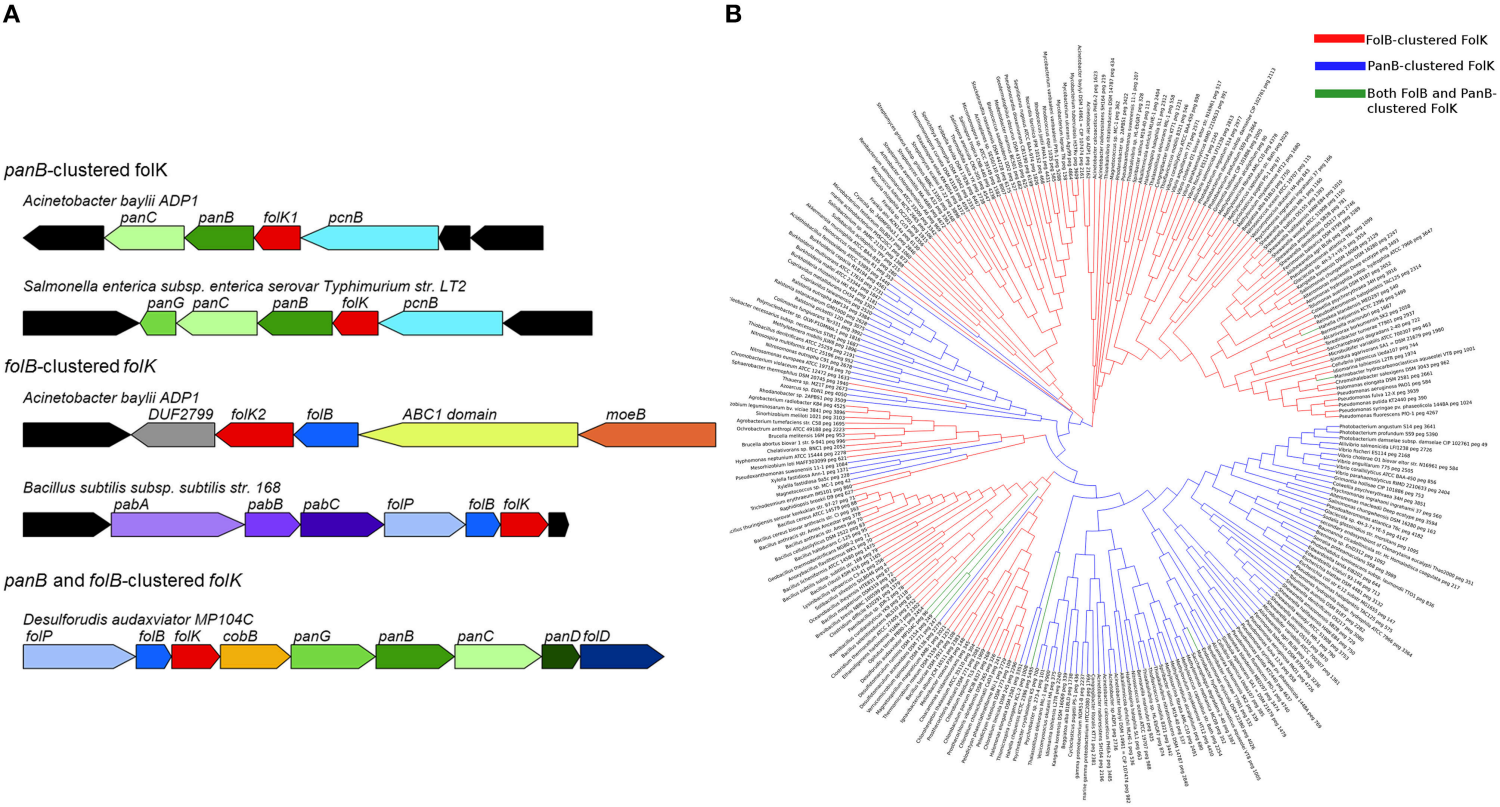
**Clustering and phylogeny of *folK*. (A)** Physical clustering of *folK* homologs with *panB* or *folB* in specific genomes. **(B)** A phylogenetic tree of FolB-clustered FolK (red), PanB-clustered FolK (blue), and FolB and PanB-clustered FolK (green). The sequences were aligned with MUSCLE and the tree was calculated with MEGA6 using the maximum likelihood method.

Physical clustering analysis showed that in ~70% of the genomes with two copies of *folK*, each *folK* gene occurs in a particular genomic context. One copy clusters with *folB*, which encodes the preceding step in folate synthesis (Figure 1), and the other clusters with a pantothenate biosynthesis gene, *panB*, as seen in *Acinetobacter baylyi* ADP1 (Figure 2A). These clusters also reflect the gene organization in genomes that have only one *folK* gene. Thus, 25% of the organisms in SEED harbor a single *folK* gene that clusters with *folB* (e.g., *Salmonella enterica*) and 18% harbor a single *folK* gene that clusters with *panB* (e.g., *Bacillus subtilis*) (Figure 2A). Furthermore in certain genomes (e.g., *Desulforudis audaxviator*) a single *folK* gene clusters with both *panB* and *folB* (Figure 2A). To eliminate any bias introduced by overrepresentation of certain species, the analysis was repeated on a set of 981 organisms chosen for their diversity (Niehaus et al., 2015). Very similar results were obtained: 7% of the genomes contained two *folK* genes with one next to *panB* and the other next to *folB*; 20% of the genomes had a unique *folK* next to *folB*; and 11% had a unique *folK* next to *panB*. Consistent with the functional link between the *folK* and *panB* genes implied by their clustering in bacteria, the FolK and PanB enzymes localize to the same subcellular compartment (the mitochondrial matrix) in plants and yeast (Güldener et al., 2004; Ottenhof et al., 2004; Perocchi et al., 2006; Gerdes et al., 2012). To explore structural, and potential functional, differences between the products of *folK* genes clustered with *panB* or *folB* genes, we aligned ~270 FolK sequences that were chosen from a diverse set of prokaryotes that included organisms with *folK* duplications. In order to produce a higher quality alignment, proteins were kept only if their lengths were within 0.25 standard deviations of the average length of the set, or if there was another FolK encoded in the same genome. Sequence logo comparisons showed differences in enrichments for specific amino acids at given positions between the FolK proteins whose genes physically cluster with *panB* genes and those that cluster with *folB* (Figure S1). These amino acid differences between the two groups were confirmed by phylogenetic analyses on the same set of sequences as that for the majority of FolK proteins; the phylogenetic clustering broadly matched the physical clustering (Figure 2B). The separation is not perfect, however, so these signatures do not necessarily point to a functional divergence between the two FolK groups.

In order to test whether, from genomes with two copies of *folK*, both have 6-hydroxymethyl-7,8-dihydropterin pyrophosphokinase activity *in vivo*, the *folK1* and *folK2* genes of *A. baylyi* ADP1 were tested for the capacity to complement the dT auxotrophy a Δ*folK* strain of *E. coli.* The *folK* homolog found next to *panB* (*folK1*, ACIAD3062) robustly complemented the dT auxotrophy, whereas the one next to *folB* (*folK2*, ACIAD2407) did not (Figure S2A). This fits with gene essentiality data for *A. baylyi* showing that *folK1* is essential and that *folK2* is not (de Berardinis et al., 2008). The *folK* genes that cluster with *panB* may thus be taken to be functional. We were surprised by these results as many organisms have just one *folK* that clusters with *folB* and must therefore be active. Analysis of the FolK multiple alignments revealed that two critical residues that interact with Mg^2+^, ATP, and substrates (R92, F123 in *E. coli* FolK numbering Blaszczyk et al., 2000) are not conserved in the *A. baylyi* FolK that clusters with *folB* (Figure S2B). This was an exception of the *Acinetobacter* clade because in most organisms with duplicated FolKs, all catalytic residues were conserved in both copies (data not shown).

### A hypothesis linking the *panB*-*folK* association to folate damage and its repair

**Figure 3:**
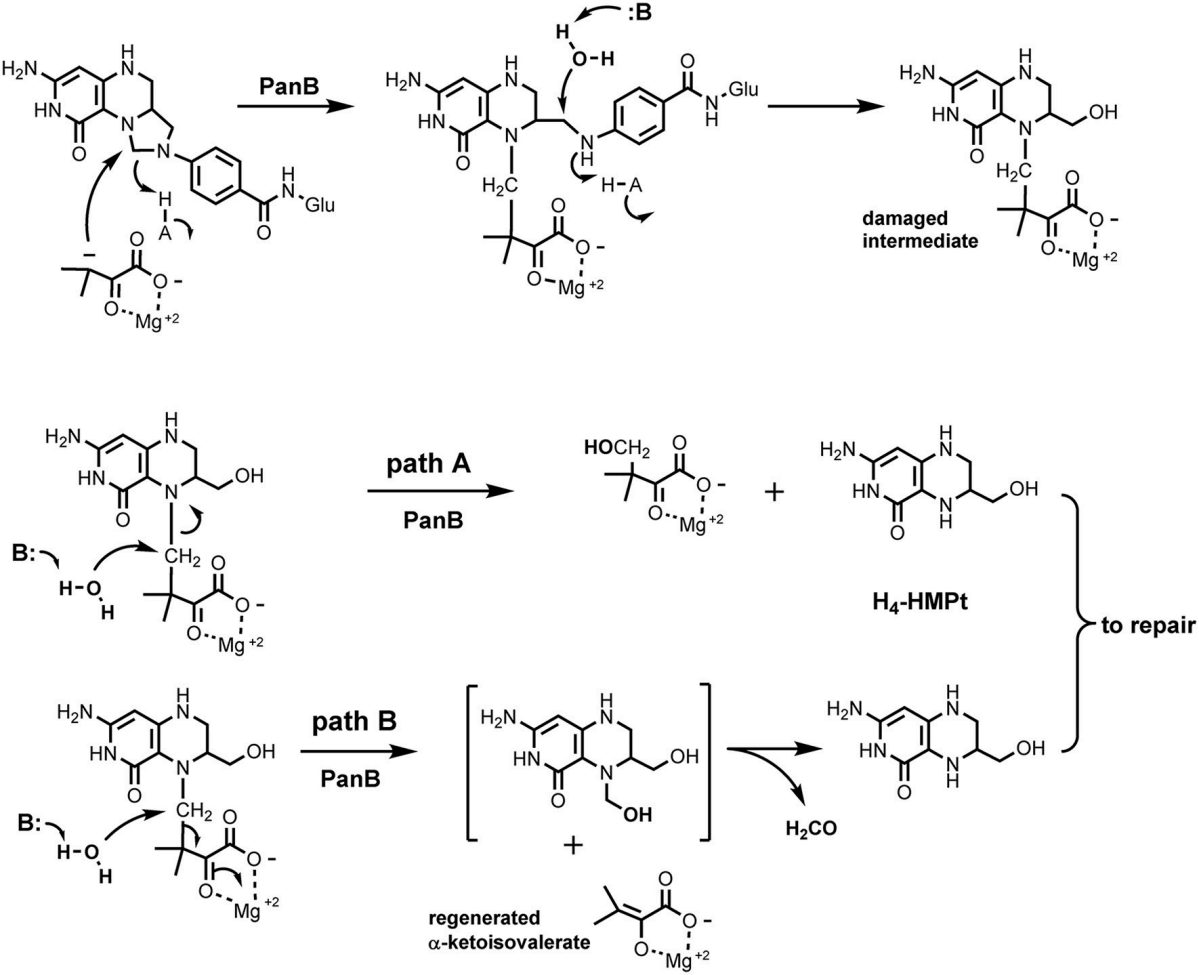
**Proposed side activity of PanB**. PanB initiates normal carbon transfer to α-ketoisovalerate to form the initial adduct which then subsequently undergoes aberrant hydrolytic attack to cleave the glutaminyl-p-ABA group from the pterin system. The resulting adduct can cleave by path A which generates 2-dehydropantoate and H_4_-HMPterin. In path B, hydrolytic cleavage releases unchanged α-ketoisovalerate and produces the bis-hydroxymethyl intermediate that spontaneously decomposes to formaldehyde and H_4_-HMPterin. Subsequent redox steps would convert the H_4_-HMPterin to H_2_-HMPterin for re-entry into the biosynthetic pathway.

The *panB* gene encodes the CH_2_-THF-dependent enzyme ketopantoate hydroxymethyltransferase (EC 2.1.2.11). This suggested an explanation for the observed association between *panB* and *folK* based on the proposed reaction mechanism of PanB. Powers, Snell, and coworkers first purified this enzyme and showed that it is a class II aldolase (i.e., metal requiring) (Powers and Snell, 1976; Teller et al., 1976). In the proposed mechanism, PanB initiates the normal reaction sequence by forming an adduct between α-ketoisovalerate and CH_2_-THF; the adduct is then resolved by hydrolytic attack to generate THF and the product 2-dehydropantoate. We hypothesized a damage side-reaction in which the adduct first undergoes hydrolytic attack to liberate the pABA-Glu moiety (Figure 1), with concomitant formation of a hydroxymethyl group on the pterin/α-ketoisovalerate adduct (Figure 3). This adduct then undergoes a second hydrolytic attack, with two possible outcomes, both involving production of 2-amino-4-hydroxy-6-hydroxymethyl-7,8-tetrahydropteridine (H_4_-HMPterin). In path A, the other reaction product is 2-dehydropantoate; in path B, α-ketoisovalerate is regenerated along with an unstable bis-hydroxymethylpterin, which spontaneously loses formaldehyde to give H_4_-HMPterin. The H_4_-HMPterin formed by either path could re-enter the folate pathway via FolK after spontaneous oxidation to the dihydro form (Figure 1), or possibly directly. We therefore made genetic tests of the hypothesis that PanB damages CH_2_-THF and that the resulting pterin moiety is recycled to folate via FolK.

### Genetic and metabolic evidence favor a role of PanB in THF damage

If a side-reaction of PanB cleaves CH_2_-THF (Figure 3), then overexpressing *panB* should make wild type *E. coli* more sensitive to antifolate drugs that deplete folate pools, such as sulfathiazole (a pABA analog) and trimethoprim (a dihydrofolate reductase inhibitor).

**Figure 4:**
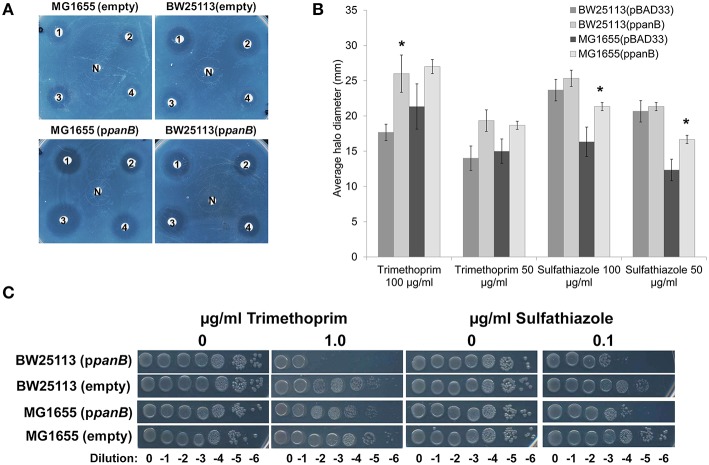
**Antifolate sensitivity (A) Overnight cultures of *E. coli* BW25113 and MG1655 carrying pCA24N::*panB* (p*panB*) or pBAD33 (empty) were diluted 100-fold and spread onto M9-glucose agar medium containing chloramphenicol and IPTG (0.1 mM)**. Twenty microliter of (1) sulfathiazole at 100 μg/ml, (2) sulfathiazole at 50 μg/ml, (3) trimethoprim at 100 μg/ml, (4) trimethoprim at 50 μg/ml, or (N) media with no antibiotic were spotted onto filter disks. **(B)** Diameter of the halo inhibition were measured for three biological replicates of each and the average and standard deviation of each were calculated.^*^ indicates significant difference from empty vector control (*p* > 0.05 determined by 2-tailed Student's *t*-test). **(C)** Serial dilutions of each strain were spotted onto M9-glucose agar plates with chloramphenicol and IPTG with trimethoprim (0 or 1 μg/ml) or sulfathiazole (0 or 0.1 μg/ml). *E. coli* overexpressing *panB* were more sensitive to both folate inhibitors than *E. coli* with empty vector control.

The effect of overexpressing the *panB* was tested by transforming either *E. coli* strain BW25113 or MG1655 with the ASKA clone expressing *panB* under control of the *lac* promoter (p*panB*) or with an empty vector (pBAD33) control. Sensitivity to trimethoprim and sulfathiazole was assessed for each strain by measuring the halos of inhibition of different concentrations of each drug. The strains overexpressing *panB* were more sensitive to both trimethoprim and sulfathiazole resulting in larger zones of inhibition (Figures 4A,B). *E. coli* MG1655 showed a greater increase in sensitivity to sulfathiazole when overexpressing *panB* than BW25113; conversely, BW25113 overexpressing *panB* had a greater increase in sensitivity to trimethoprim than MG1655. To confirm the exacerbated sensitivity, ten-fold serial dilutions of late log-phase cultures were spotted onto M9-glucose agar plates containing chloramphenicol, IPTG, and various levels of trimethoprim or sulfathiazole (Figure 4C). Trimethoprim at 1 μg/ml did not affect growth of the empty-vector control cells but reduced growth of the BW25113 *panB*-overexpressing cells by a factor of 10^5^. Similarly, overexpressing *panB* made *E. coli* 10^2^-fold more sensitive to 0.1 μg/ml sulfathiazole. As observed with the inhibition halos, the trimethoprim sensitivity of MG1655 overexpressing *panB* was less drastic than BW25113, however, the sulfathiazole sensitivity of both *panB*-overexpressing strains was similar.

To assess whether deleting *panB* affected sensitivity to antifolates, BW25113 Δ*panB* was acquired from the Keio collection (Baba et al., 2006). Ten-fold serial dilutions of wild-type and mutant were spotted onto M9 glucose supplemented with pantothenate and various levels of sulfathiazole or trimethoprim. Deletion of *panB* did not alter the sensitivity to either antifolate (data not shown).

Growth phenotypes can be caused by very indirect effects, as shown in recent studies on mutants affected in vitamin B_6_ damage that propagates to CoA and folate metabolism (Flynn et al., 2013; Downs and Ernst, 2015). Pterin pools were therefore analyzed in strains overexpressing or lacking *panB* to determine whether direct effects could be observed. According to our hypothesis, overexpressing *panB* will cause a buildup in cells or medium of H_4_-HMPterin and its dihydro form (which are both analyzed as the oxidized form 6-hydroxymethylpterin).

**Figure 5:**
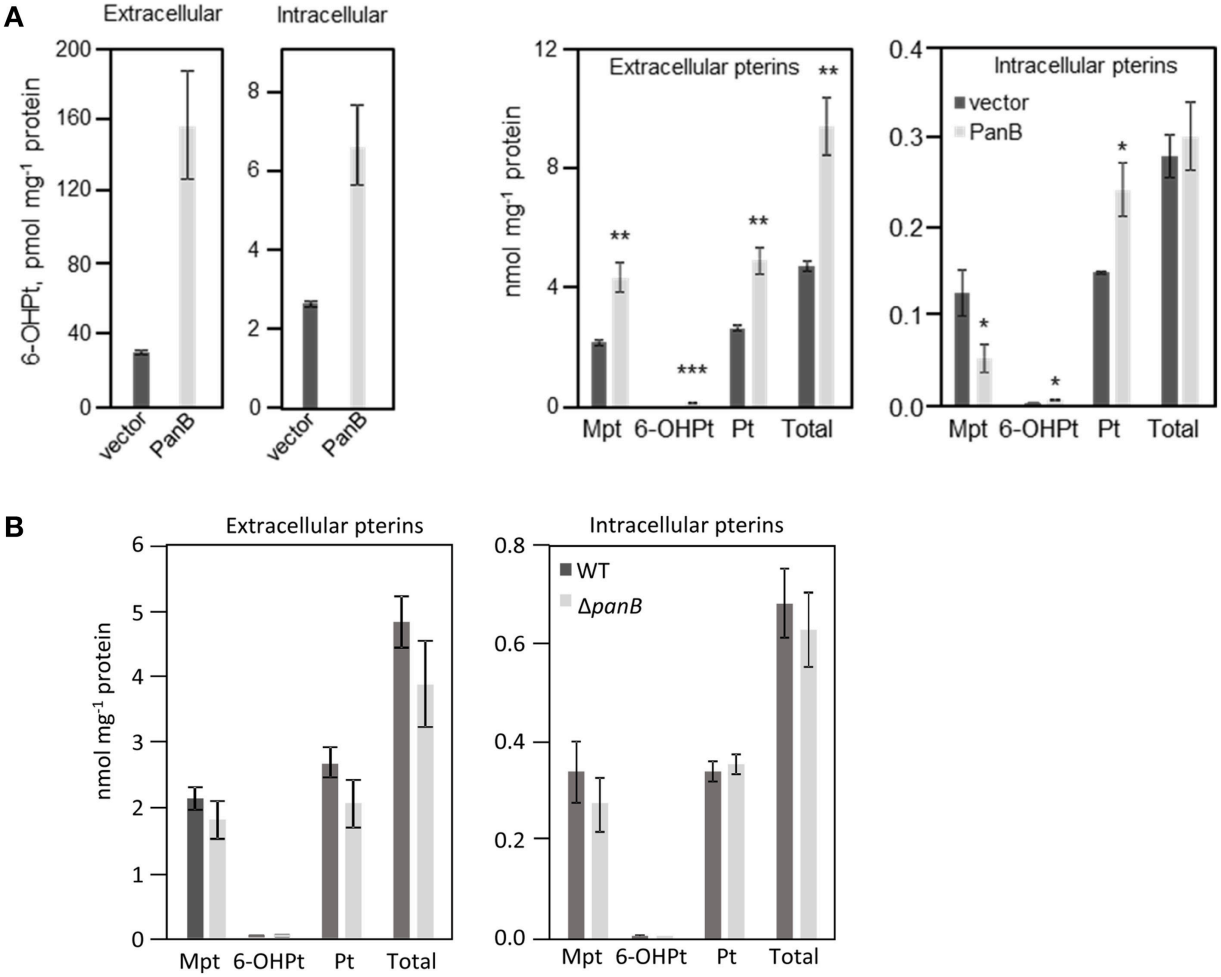
**Quantification of pterin pools in *E. coli* cells over- or underexpressing PanB**. **(A)** Quantitation of intra- and extracellular pterins extracted from *E. coli* wild type MG1665 harboring pUC19 (vector) or overexpressing *panB* (PanB). Strains were grown in liquid M9 medium plus 0.4% glucose to an A_600_ of 1.0. Cultures were induced by the addition of 0.5 mM IPTG at an A_600_ of 0.3. Cell extracts and media were oxidized before analysis by HPLC to convert di- and tetrahydropterins to their fluorescent aromatic forms. Pterin contents of both extacts and media are expressed per unit of protein in the cells from which they came. Data are means and standard errors from three biological replicates, and were subjected to a *t*-test. Differences in pterin content between vector control and the *panB* overexpressing strains that are significant at *P* < 0.05, < 0.01, or < 0.001 are respectively marked by one, two, or three asterisks. Mpt, monapterin; Pt, pterin; 6-OHPt, 6-hydroxymethylpterin. Note the 1000-fold difference in scale between the two frames on the left (which report only 6-hydroxymethylpterin levels) and the two frames on the right (which report the levels of all pterins measured). **(B)** Quantification of intra- and extracellular pterins extracted from *E. coli* wild type BW25113 and Δ*panB*::kan mutant. Strains were grown in liquid M9 medium plus 0.4% glucose and 10 μM pantothenate to an A_600_ of 1.0. Samples were treated and analyzed as in **(A)**.

Overexpression of *panB* (as a pUC19 construct) in *E. coli* MG1655 indeed led to accumulation of 6-hydroxymethylpterin in both medium (five-fold) and cells (three-fold) (Figure 5A). Also, the total pterin content of the medium doubled in cultures overexpressing *panB*; the total intracellular content remained same as the control (Figure 5A). The ratio of pterin to monapterin was 4.5 in cells overexpressing *panB* compared to 1.2 in control cells (Figure 5A). Note that tetrahydromonapterin (analyzed as monapterin) is known to be a major *E. coli* pterin (Pribat et al., 2010) and that pterin is a breakdown product of both tetrahydromonapterin and 6-hydroxymethyldihydropterin (Forrest and Van Baalen, 1970; Davis et al., 1988). The data for overexpression of *panB* are thus consistent with a damage hypothesis. The deletion of *panB* had no impact on pterin pools (Figure 5B).

**Figure 6:**
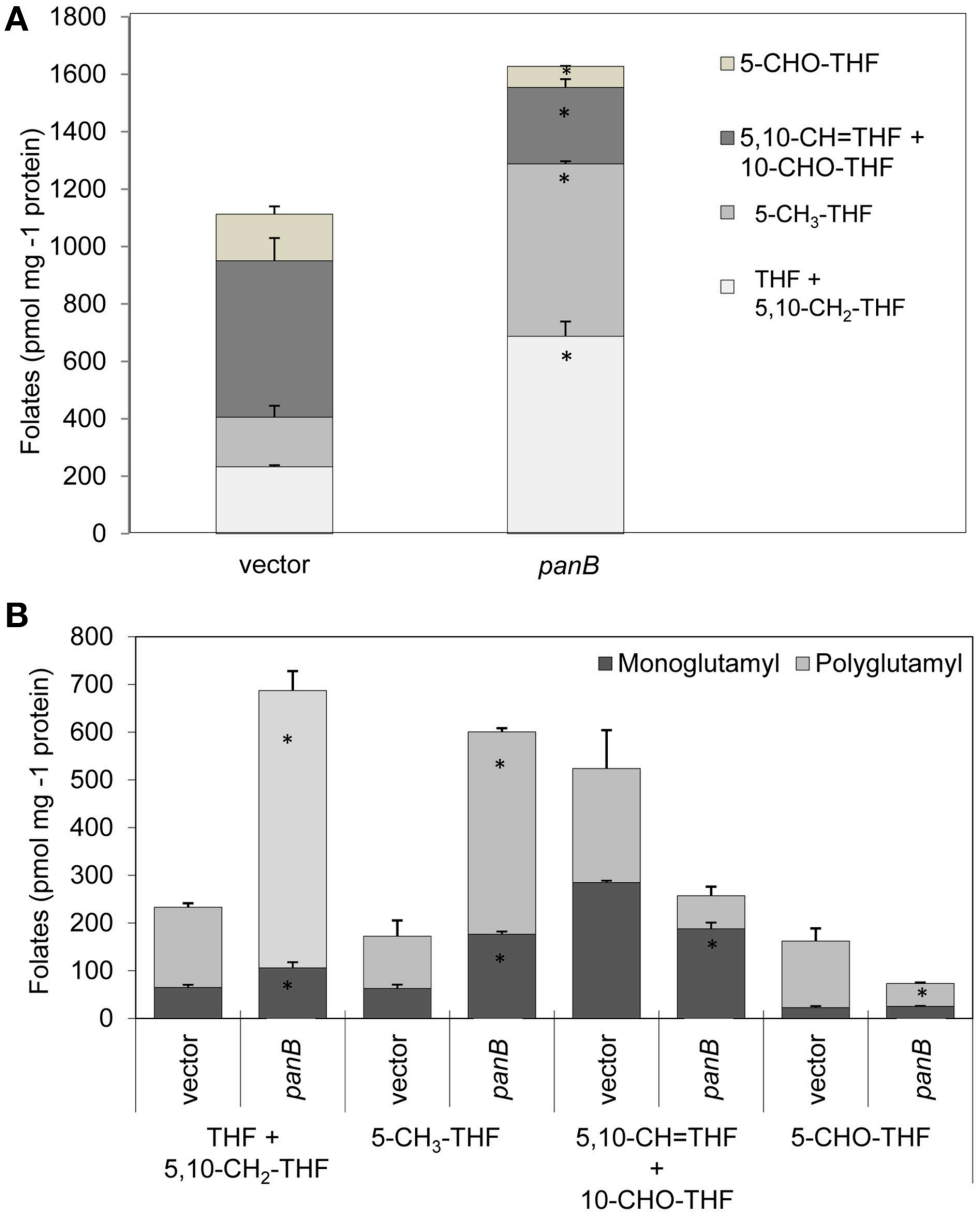
**Effect of PanB overexpression on folate levels in *E. coli* cells**. Wild type *E. coli* cells overexpressing *panB* or harboring empty vector (control) were grown as in Figure 5A. **(A)** Total folates observed. The total folate level in cells overexpressing PanB was significantly different (^*^p < 0.05) from that in the vector control. **(B)** Total folates observed in mono- and polyglutamyl form. Abbreviations: 5,10-CH_2_-THF, 5,10- methylenetetrahydrofolate; 5-CH_3_-THF, 5-methyltetrahydrofolate; 5,10-CH = THF, 5,10-methenyltetrahydrofolate; 10-CHO-THF, 10-formyltetrahydrofolate; 5-CHO-THF, 5-formyltetrahydrofolate. Small amounts of 10-formyldihydrofolate (which forms during extraction from 10-formyltetrahydrofolate) were added to the 10-formyltetrahydrofolate pool.

Folate pools were also analyzed in the strain overexpressing *panB*. As shown in Figure 6A, a 46% increase in total folates was observed, contributed by substantial increases in 5-methyltetrahydrofolate and in THF/CH_2_-THF (which are both analyzed as THF) that were partially offset by decreases in 5-formyl-THF and 5,10-methenyl-THF/10-formyl-THF (which are analyzed together). The folate pools that increased were largely polyglutamyl forms (Figure 6B), which are generally more metabolically active than monoglutamyl forms (Suh et al., 2001; Green and Matthews, 2007). The increase in total folate content appeared counter-intuitive as a folate-cleaving reaction of PanB might be expected to deplete folates.

The observed increase in folate pools upon overexpression of *panB* suggested a potential regulation of the folate genes, particularly those involved in pterin synthesis. Gene expression of *folK, folB, folE*, and *panB* was analyzed by quantitative PCR in *E. coli* MG1655 overexpressing *panB* (as the pCA24N construct). Upon IPTG induction, the expression of *panB* increased over *E. coli* MG1655 harboring empty vector by up to 6000-fold. When *panB* induction was 6000-fold, the expression of *folK, folB*, and *folE* increased by about 10-fold (Figure S3). With the level of *panB* induction achieved was lower (500-fold), however, the expression of all three folate genes was essentially unaffected (not shown). Variation in induction of *panB* presumably reflects the stochastic nature of induction, as documented for the *lac* promoter both experimentally and through modeling (Novick and Weiner, 1957; Elowitz et al., 2002; Stamatakis and Mantzaris, 2009).

### Metabolic modeling reconciles the experimental evidence with the PanB damage model

A kinetic model (Figure S4) of the folate synthesis pathway was constructed to explore potential mechanistic explanations for the increase in folate pools observed when PanB is overexpressed. The model includes the last four steps of THF synthesis, the GlyA reaction, which generates CH_2_-THF, and the proposed folate-cleaving side-reaction of PanB. The GlyA reaction is modeled to operate near equilibrium, while the four preceding reactions are all modeled as irreversible and unsaturated (meaning reaction rates are proportional to reactant concentrations). This is consistent with the kinetic and thermodynamic data available for these reactions (Jankowski et al., 2008; Chang et al., 2015). The proposed PanB side reaction is also modeled as irreversible and unsaturated. A sensitivity analysis of the model (see Methods) reveals that model results are not impacted greatly by assumptions regarding the saturation states of the included enzymes. However, the near-equilibrium state of the GlyA reaction was found to be very important for controlling the steady state ratio of THF and CH_2_-THF predicted by the model.

Kinetic parameters and metabolite concentrations were selected for the model to fit physiological conditions and replicate the wild-type concentrations measured for H_2_-HMPt and THF (Figure S4). The model also replicated the net production rates predicted for THF compounds in wild-type cells based on measured growth rates (1.66e-7 mmol ml^−1^ cytoplasm s^−1^). While measured values are not available for many of the metabolite concentrations and kinetic parameters in the model, these parameters are restricted by the need to replicate experimental observations. Once complete, we simulated the model under wild-type conditions, demonstrating that the steady-state predicted by the model was consistent with all the experimental observations.

**Figure 7:**
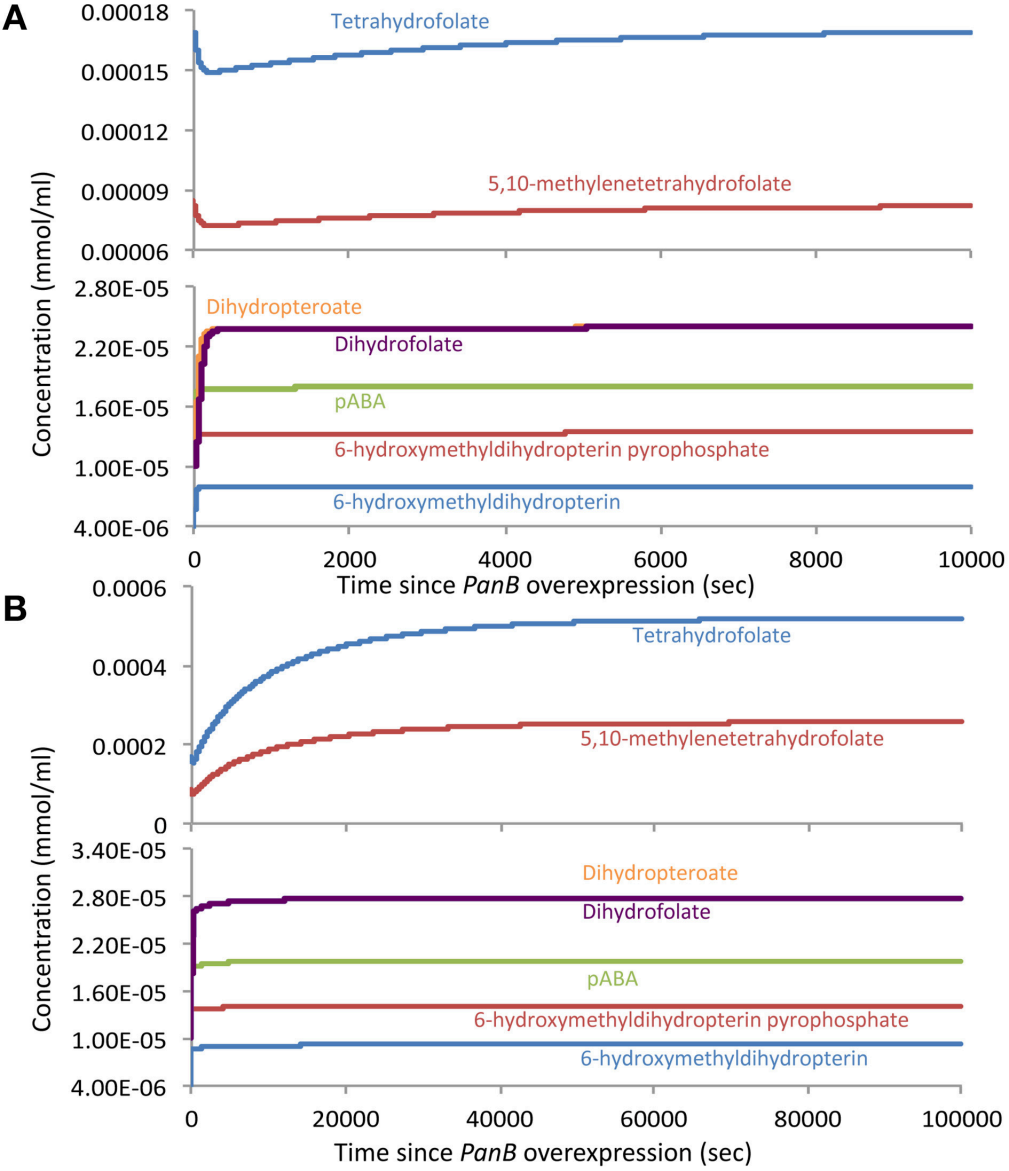
**Model-predicted changes in the concentrations of folates and their precursors in response to overexpression of PanB. (A)** The response when only the *V*_max_ of the PanB folate-cleaving reaction is increased, leaving other model parameters the same. **(B)** The response when both the PanB *V*_max_ and the external flux into 6-hydroxymethyldihydropterin are increased at the same time.

Next, we simulated the overexpression of PanB by increasing only the kinetic constant of the mass-action kinetic equation for the PanB folate-cleaving reaction (which integrates the enzyme concentration of PanB), leaving all other model parameters the same. In this simulation, all metabolite concentrations started at the steady-state values predicted from the wild-type model (Figure 7A). Immediately, the CH_2_-THF (and THF) concentration began to drop due to the larger flux through the PanB side reaction. This led to a rise in the concentrations of H_2_-HMPt (and its pyrophosphate, the PanK reaction product), which in turn led to a rise in the flux through the THF synthesis pathway, leading to the slow recovery of the THF concentration back to—but not exceeding—the wild-type values. Thus, this simulation successfully replicates the rise in H_2_-HMPt concentration that accompanied the overexpression of PanB in our experiments, but it fails to replicate the rise in THF concentrations, meaning the overexpression of PanB alone cannot explain all experimental observations.

We therefore hypothesized that the initial drop in THF concentration predicted when PanB is overexpressed triggers induction of the enzymes leading into the THF synthesis pathway, thereby increasing the flux to folates and more rapidly restoring folate concentration. We tested this hypothesis by repeating the previous simulations, but this time increasing the external flux into H_2_-HMPt and pABA at the same time as we increase the kinetic constant of the mass-action kinetic equation for PanB kinetic constant. In this scenario, we still see an initial dip in THF concentrations, which accompanies the rise in H_2_-HMPt, but this time, the THF concentrations recover more rapidly and ultimately rise beyond the wild-type levels and reach new steady-state values that closely resemble those observed in the PanB overexpression experiments (Figure 7B). The analysis of expression levels the *folEBK* by quantitative PCR (Figure S3) indicate that an increase in H_2_-HMPt biosynthesis can indeed occur when *panB* is highly overexpressed.

A remaining issue is whether our experimental observations can be reproduced by a kinetic model that lacks a PanB-mediated folate cleavage reaction. If one models the FolK reaction to be in a saturated state with appropriately tuned kinetic parameters, the observed changes in metabolite concentration can be replicated to co-occur with a rise in flux through the folate pathway. However, this scenario does not explain why the folate pathway flux rises with the induction of PanB, nor does it explain the direct link between the THF and H_2_-HMPt concentrations implied by the experimental data. A model that includes the PanB folate-cleaving reaction thus accounts more fully for the observations than one that does not.

## Discussion

This work illustrates the power of comparative genomics approaches to uncover novel damage reactions and repair mechanisms (Linster et al., 2013). Physical clustering combined with gene duplication clues in association with analysis of the biochemical literature led us to infer that PanB damages the THF molecule as a side reaction. In the course of normal activity, PanB catalyzes formation of an adduct between α-ketoisovalerate and CH_2_-THF (Figure 3). In order to release pABA-Glu from the complex, we hypothesize that PanB provides a suitably disposed general acid to deliver a proton to the pABA nitrogen, simultaneous with general base catalyzed attack of water at the methylene carbon to release pABA and generate the 6-hydroxymethyl group.

Possible path A of the side reaction involves an attack of water to release 2-dehydropantoate and directly produce H_4_-HMPterin. Possible path B involves hydrolytic attack on the bridging methylene carbon between the pterin ring and the α-ketoisovalerate adduct. This displacement reaction by water is reasonable given the stabilization of the enolate leaving group as the Mg^2+^ complex, and possible general base catalysis to assist the attack of water. The resulting N5 hydroxymethyl group is a hemiaminal and should equilibrate to liberate free H_4_-HMPterin and formaldehyde. One question remains open, however, as the product of this side reaction is H_4_-HMPterin, but the dihydro form is the normal FolK substrate. Thus, either there is an oxidation step, most likely spontaneous (Davis et al., 1988) before FolK acts, or FolK (and the subsequent folate pathway enzymes FolP and FolC) can act on the tetrahydro forms of their substrates instead of the dihydro forms—in which case DHFR/FolA would be dispensable for this salvage reaction. Analysis of the structures of FolK, FolP, and FolC with substrates (PDB codes 1Q0N, 2DZB, 1W78, and Figure S5) suggests that these three enzymes would not discriminate between the di- and tetrahydro forms of their substrates and that H_4_-HMPterin could be recycled.

Overexpression of PanB caused expected and unexpected effects. Expectedly, levels of 6-hydroxymethylpterin and total pterins increased. Unexpectedly, so did folate levels. However, as kinetic modeling showed, the folate increase can be accounted for by a very reasonable assumption, viz. that depletion of certain folates (or conceivably the buildup of folate damage products) leads to upregulation of the folate pathway. he observed increase in folate production appears to be the only case so far reported of a folate pathway regulatory response to a physiological perturbation (Tran and Nichols, 1991; Green and Matthews, 2007). The scale of the upregulation of the pterin branch of the folate pathway—a doubling (Figure 5A)—is particularly striking, as is the loss to the medium of most of the extra pterin production. It should be noted that some cell lysis was observed in cells overexpressing *panB*, which could account for a portion of the observed increase in extracellular pterins. However, it was previously observed that, in wild type *E. coli*, folate synthesis accounts for <20% of the pterin moieties produced, most of which are exported to the medium (Pribat et al., 2010). Our present data agree with this observation (Figures 5A, 6A), and further show that the increase in total folate production elicited by PanB overexpression (0.5 nmol mg^−1^ protein) was almost ten-fold less than the increase in pterin production (4.7 nmol mg^−1^ protein), all of it found in the medium. Thus, an increase in intracellular folate cleavage triggers a doubling of pterin production, most of which flows not to folate synthesis but out into the medium.

The expanded folate pool in *E. coli* cells overexpressing *panB* raises the question of how this observation can be reconciled with the antifolate sensitivity phenotypes of these cells (Figure 4B). One possibility is that pABA-Glu recycling is inefficient, so that an increased flux to folates depletes the pABA pool, which increases sulfathiazole sensitivity. This scenario is supported by the findings that pterin overproduction in tomato fruit increased the flux to folates but caused pABA depletion (Díaz de la Garza et al., 2004), and that pABA supplementation increased folate production in *Lactococcus lactis* (Sybesma et al., 2003). Another possibility is that accumulated pterins competitively inhibit dihydrofolate reductase, and hence potentiate the action of trimethoprim. In support of this possibility, bacterial and mammalian dihydrofolate reductases are known to bind (and inefficiently reduce) certain dihydropterins (Armarego et al., 1980; Matsuura and Sugimoto, 1981).

A role for PanB in THF damage could explain several long-standing observations. First, it has been shown that PanB is a limiting step in pantothenate synthesis (Rubio and Downs, 2002); PanB levels could have been kept low to avoid damage. Second, there is evidence that folate and pantothenate pathways cross-connect. In *Bacterium linens* str. 456, pABA was able to substitute for the pantothenate requirement and vice versa (Purko et al., 1953). Each compound was shown to enable the synthesis of the other (the pABA analysis in this study involved an acid hydrolysis step and bioassay of pABA, so that folate was probably the main source of pABA). The pABA (folate) requirement for pantothenate synthesis was subsequently explained by the discovery of the folate-dependent ketopantoate hydroxymethyltransferase PanB. The pantothenate requirement for “pABA” synthesis appears never to have been explained. This pantothenate requirement cannot be explained by a CoA-dependent step in chorismate or pABA synthesis. A simple possibility is that *B. linens* str. 456 has a PanB defect that is rescued by a high folate concentration, and that supplying pABA boosts folate content and hence pantothenate synthesis, i.e., there is no defect in folate synthesis. A more complex possibility is that *B. linens* str. 456 has a FolP defect that can be corrected by supplementary pABA, that FolP and FolK are coupled, and that in the absence of supplemental pABA, 6-hydroxymethyldihydropterin pyrophosphate accumulates and negatively regulates (Mouillon et al., 2002) the FolK reaction so that 6-hydroxymethyldihydropterin accumulates, favoring spontaneous loss of the side-chain and so further compromises folate synthesis and hence PanB activity.

It would obviously be of interest to test PanB for a folate-cleaving side activity *in vitro*. There are, however, major difficulties with this type of experiment. First, THF and CH_2_-THF are highly labile folates and readily undergo C9-N10 bond cleavage in physiological conditions (Wilson and Horne, 1983), so that the background cleavage rate against which to detect a minor activity of PanB would be high. The half-life of free THF, for example, is only about 40 min at physiological pH (Suh et al., 2001). Reduced folates are greatly stabilized against spontaneous cleavage *in vivo* by binding to enzymes and other folate-binding proteins (Suh et al., 2001), but attempting such stabilization *in vitro* would necessarily sequester folates away from PanB. Second, CH_2_-THF is in spontaneous equilibrium with THF and formaldehyde (Blakley, 1959) so that its use as a substrate entails the presence of at least a low level of formaldehyde, which reacts readily with proteins, blocking reactive residues (Metz et al., 2004). Finally, there is the concern that, like some other side-reactions (Downs and Ernst, 2015), the folate-cleaving reaction could depend so much the cellular context that it can only be investigated in a complex system.

## Author contributions

VC and AH designed the study and supervised the experimental studies, CH and AH created and analyzed the kinetic model, VC performed the bioinformatic analysis with the help of KH. JT and GH did the molecular biology and genetics experiments, OF conducted the pterin analyses. RD and CG conducted the folate analyses. NH proposed the biochemical mechanisms. VC, AH, NH and CH wrote the manuscript and all authors edited it.

## Funding

This work was funded by US National Science Foundation (NSF) grants MCB-1153413.

### Conflict of interest statement

The authors declare that the research was conducted in the absence of any commercial or financial relationships that could be construed as a potential conflict of interest.
