# Supplementary material for: Experimental and Metabolic Modeling Evidence for a Folate-Cleaving Side-Activity of Ketopantoate Hydroxymethyltransferase (PanB)
Source: Front Microbiol. 2016 Mar 31;7:431. doi: 10.3389/fmicb.2016.00431 (PMC4814558; doi:10.3389/fmicb.2016.00431)
Supplement: Supplementary file 1 [file Table1.DOCX]

Table S1. Primers used for RT-qPCR

| Name | Sequence 5’ to 3’ |
| --- | --- |
| folK-RT-F | GCTGGAAACCTCTCTTGCAC |
| folk-RT-R | CGGTCAGGCGTTCAGTATTT |
| folB-RT-F | GGATTGCCTCAGTTACGCTG |
| folB-RT-R | CCTGGCTTGCTGAGTTTGAT |
| folE-RT-F | CACTCAGTAAAGAAGCGGCC |
| folE-RT-R | TCAGCCAGGTCGAGATTCAG |
| panB-RT-F | CACGCCGGAACAAGCCTTCG |
| panB-RT-R | CCTGAACTTTGTAGCCACCGA |
| rssA-RT-F | CTTGGAGGTTGTGCCCTTGA |
| rssA-RT-R | GGTAAGGTTCTTCGCGTTGC |
| rpoA-RT-F | CGGCACAATCGATCCTGAAG |
| rpoA-RT-R | GCAGAGCGGACAGTCAATTC |
