# Supplementary material for: Experimental and Metabolic Modeling Evidence for a Folate-Cleaving Side-Activity of Ketopantoate Hydroxymethyltransferase (PanB)
Source: Front Microbiol. 2016 Mar 31;7:431. doi: 10.3389/fmicb.2016.00431 (PMC4814558; doi:10.3389/fmicb.2016.00431)
Supplement: Supplementary file 5 [file Image1.PDF]

FolK residues that are over-represented in FolK proteins whose genes cluster with *folB*

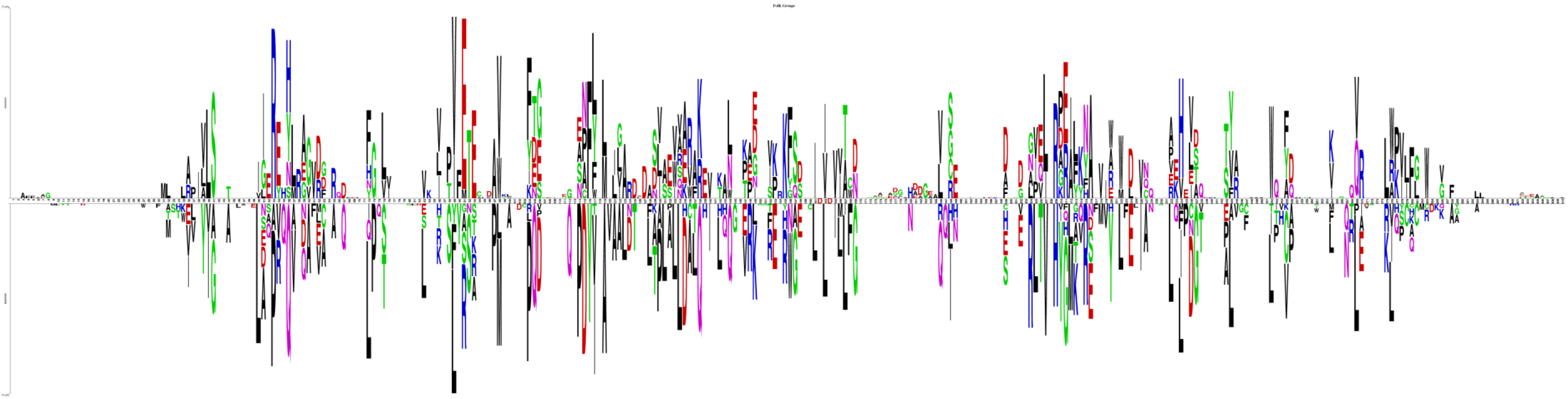

Figure S1. Comparing amino acid sequences of the two FolK subgroups. A Two Sample Logo (http://www.twosamplelogo.org/) representing the statistical significance of the relative position-specific symbol frequencies between aligned FolB-clustered FolK and PanB-clustered FolK sequences. The statistical test used was a two sample t-test. Above the midline are symbols that are over-represented in FolB-clustered sequences compared to PanB-clustered sequences, and below the midline are symbols that are under-represented FolB-clustered sequences compared to PanB-clustered sequences. The height of each symbol is proportional to the difference of relative frequencies of corresponding residues in each subgroup.

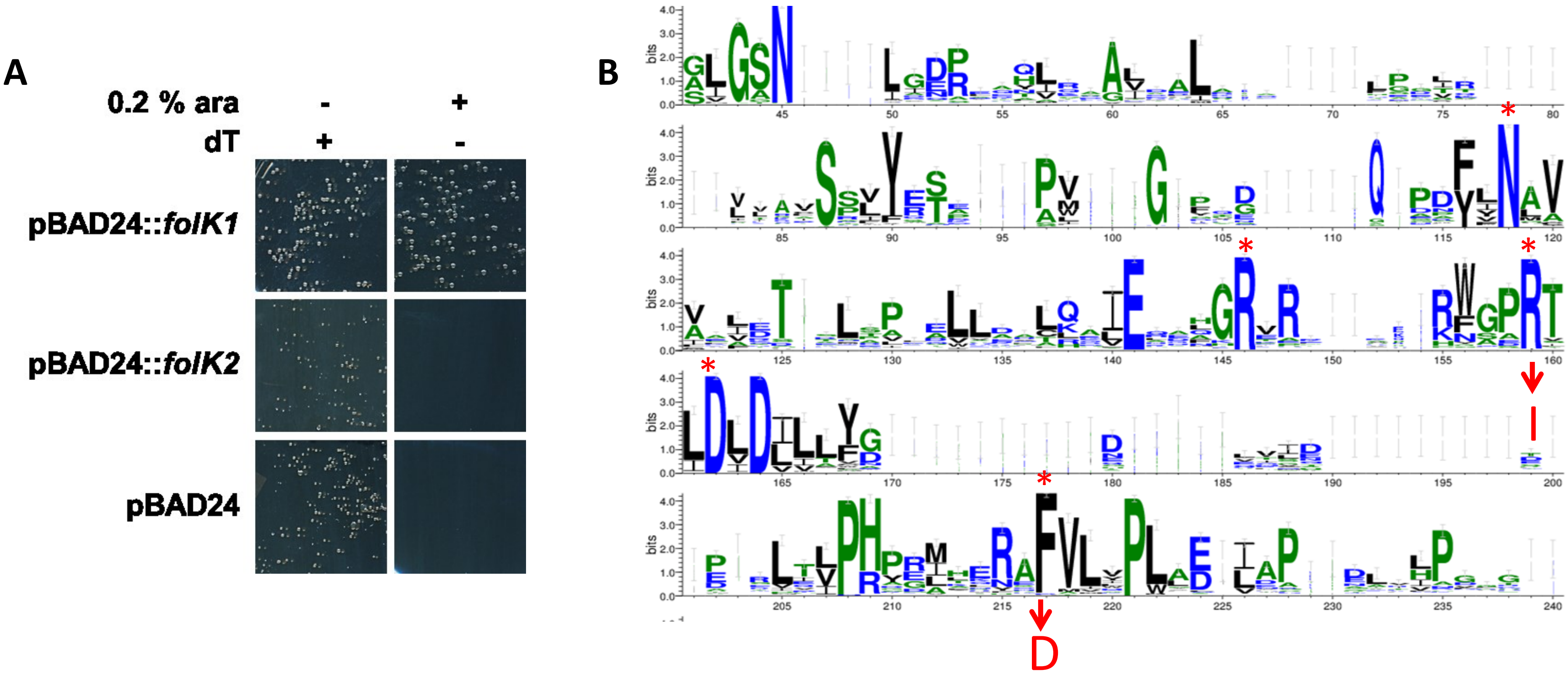

Figure S2. ***folK* complementation and conserved active site residues in FolK proteins.** (A) *E. coli* C600 Δ*folK*::*tet* transformed with plasmids containing *folK1* or *folK2* of *A. baylii* (pBAD24::*folK1*, pBAD24::*folK2*) or empty vector pBAD24 were plated onto LB agar with ampicillin with or without 0.2% arabinose and dT. C600Δ*folK*::*tet* expressing *folK1* grew on both plates after overnight incubation at 37 °C. C600Δ*folK*::*tet* expressing *folK2* or harboring empty vector grew after 48 hours on LB with dT, and did not grow on LB without dT. (B) Sequence logo derived from alignment of 272 FolK proteins. Conserved active site residues are starred, and correspond to N55, R84, R92, D95, F123 in the *E. coli* K12 protein (Uniprot: P26281). The two active site residues that are not conserved in the sequence of the *A. baylyi* ADP1 *folK* that clusters with *folB* are indicated with a red arrow with the replacement amino acid.

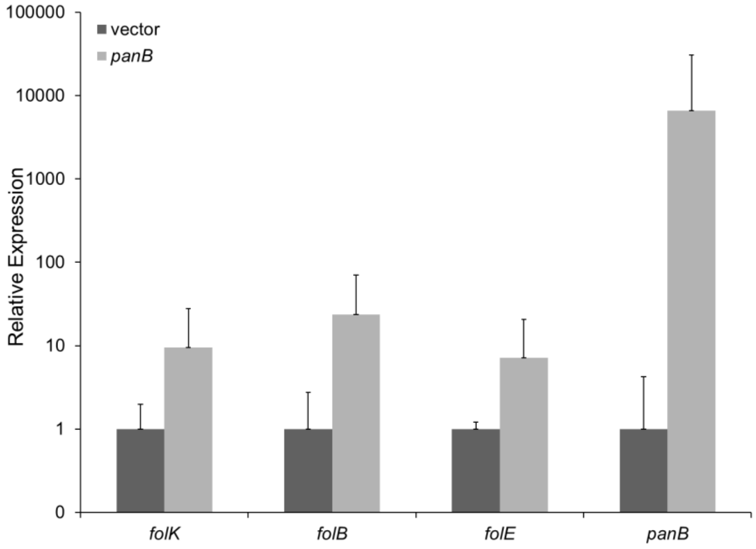

Figure S3. Relative expression levels of folate synthesis genes during *panB* overexpression. mRNA levels of *folK*, *folB*, and *folE* were determined by RT-qPCR. Each gene was normalized to two reference genes (*rssA* and *rpoA*) and the expression of each gene in *E. coli* MG1655 harboring pCA24N::*panB* (*panB*) was determined relative to the expression in *E. coli* MG1655 harboring empty vector (empty). Error bars represent the standard deviation for two technical replicates of three biological replicates in each experiment.

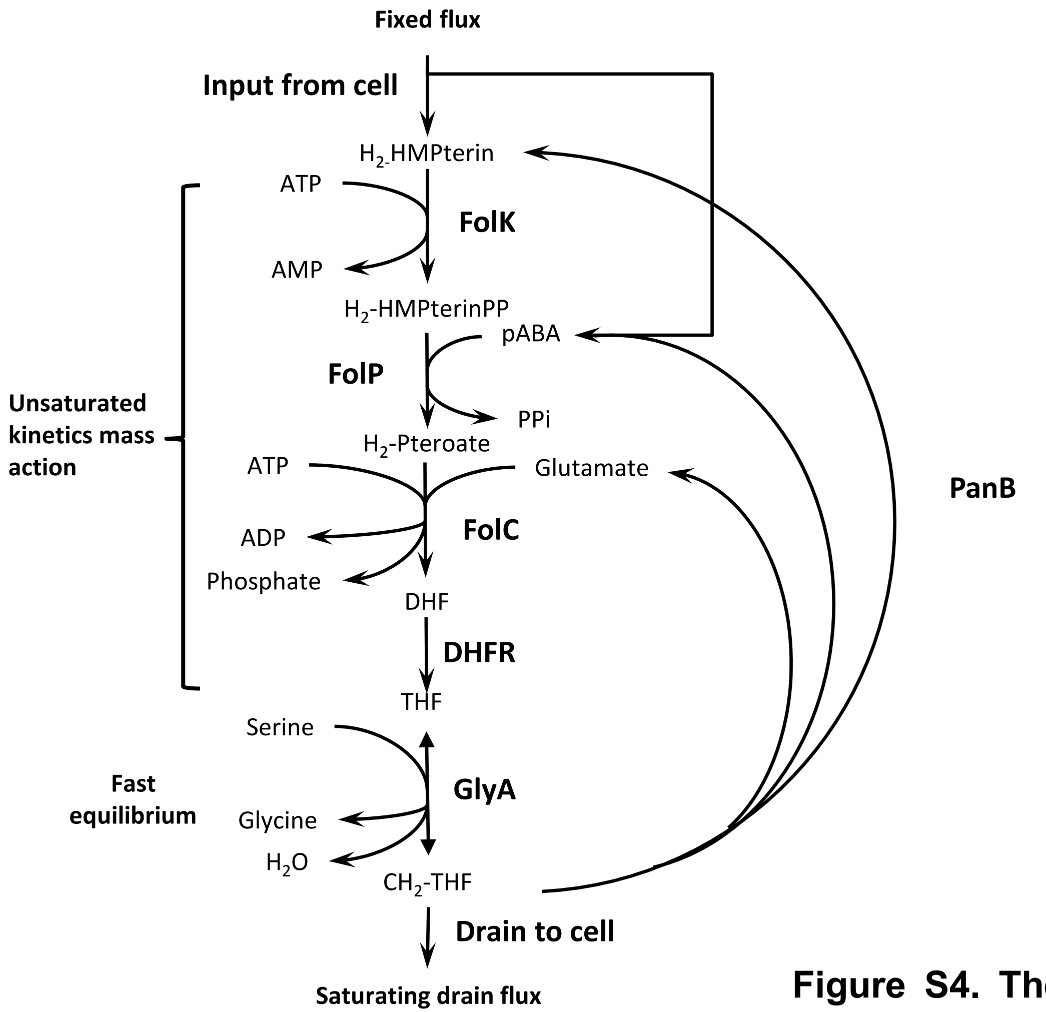

Parameter	Vector only	Plus PanB
Doubling time (min)	122	127
Total tetrahydrofolates (pmol mg^{-1} protein)	1100	1600
Total tetrahydrofolate synthesis flux (pmol mg^{-1} protein min^{-1})	9.0	12.6
5,10-methylene-THF (pmol mg^{-1} protein)*	230	690
Intracellular 2-Amino-4-hydroxy-6-hydroxymethyl-7,8-dihydropteridine (pmol mg-1 protein)	3	7
Extracellular 2-Amino-4-hydroxy-6-hydroxymethyl-7,8-dihydropteridine (pmol mg-1 protein)	30	150
*THF and 5,10-methylene-THF taken to be in equilibrium, almost all in the 5,10-methylene-THF form		

Figure S4. The kinetic model and the parameters on which it is based. The concentration parameters are derived from Figures 5 and 6. The doubling times are those observed in the experiments of Figures 5 and 6.

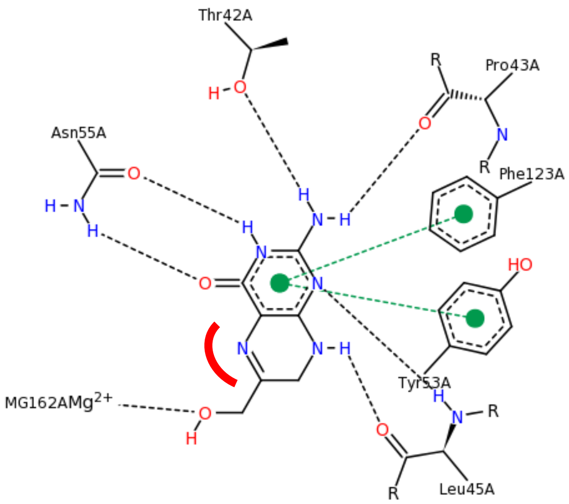

E. coli FolK in complex with H_2-HMPterin, PDB : 1Q0N

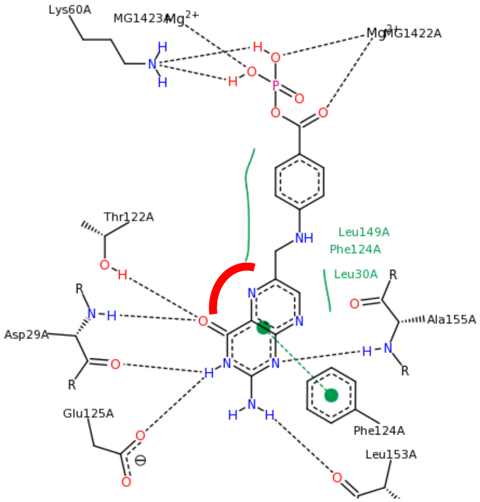

E. coli FolC in complex with DHPP and ADP, PDB: 1W78

Figure S5. Recognition of substrates by FolK, FolP and FolC enzymes. Structures of FolK, FolP and FolC and with bound substrates suggests that the three enzymes would not discriminate between the dihydro and the tetrahydro forms of the substrates. Data taken directly from PDB.

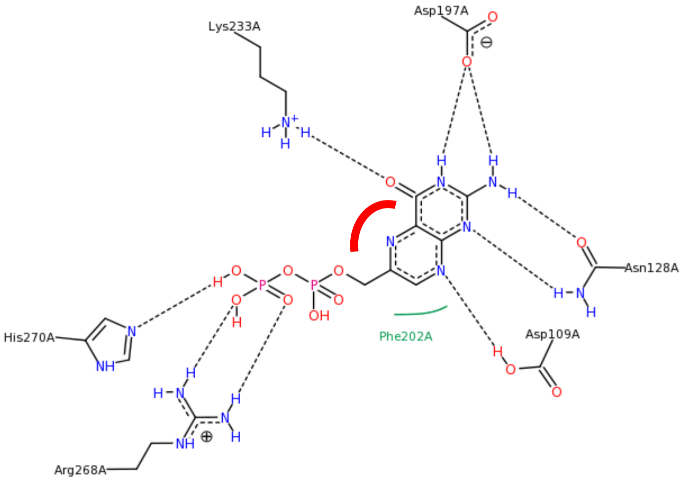

Thermus thermophilus HB8 FolP in complex with H2-HMPterinPP, PDB: 2DZB
